# The Design and Development of Potent Small Molecules as Anticancer Agents Targeting EGFR TK and Tubulin Polymerization

**DOI:** 10.3390/ijms19020408

**Published:** 2018-01-30

**Authors:** Saleh Ihmaid, Hany E. A. Ahmed, Mohamed F. Zayed

**Affiliations:** 1 Pharmacognosy and Pharmaceutical Chemistry Department, Pharmacy College, Taibah University, Al-Madinah Al-Munawarah 41477, Saudi Arabia; mfzayed25@yahoo.com; 2 Pharmaceutical Organic Chemistry Department, Faculty of Pharmacy, Al-Azhar University, Cairo 11884, Egypt; 3 Pharmaceutical Chemistry Department, Faculty of Pharmacy, Al-Azhar University, Cairo 11884, Egypt

**Keywords:** anthranilate, diamide, EGFR kinases, tubulin polymerization, anticancer activity

## Abstract

Some novel anthranilate diamides derivatives **4a**–**e**, **6a**–**c** and **9a**–**d** were designed and synthesized to be evaluated for their in vitro anticancer activity. Structures of all newly synthesized compounds were confirmed by infra-red (IR), high-resolution mass (HR-MS) spectra, ^1^H nuclear magnetic resonance (NMR) and ^13^C nuclear magnetic resonance (NMR) analyses. Cytotoxic screening was performed according to (3-(4,5-dimethylthiazole-2-yl)-2,5-diphenyltetrazolium bromide) tetrazolium (MTT) assay method using erlotinib as a reference drug against two different types of breast cancer cells. The molecular docking study was performed for representative compounds against two targets, epidermal growth factor receptor (EGFR) and tubulin in colchicine binding site to assess their binding affinities in order to rationalize their anticancer activity in a qualitative way. The data obtained from the molecular modeling was correlated with that obtained from the biological screening. These data showed considerable anticancer activity for these newly synthesized compounds. Biological data for most of the anthranilate diamide showed excellent activity with nanomolar or sub nanomolar half maximal inhibitory concentration (IC_50_) values against tumor cells. EGFR tyrosine kinase (TK) inhibition assay, tubulin inhibition assay and apoptosis analysis were performed for selected compounds to get more details about their mechanism of action. Extensive structure activity relationship (SAR) analyses were also carried out.

## 1. Introduction

**Figure 1:**
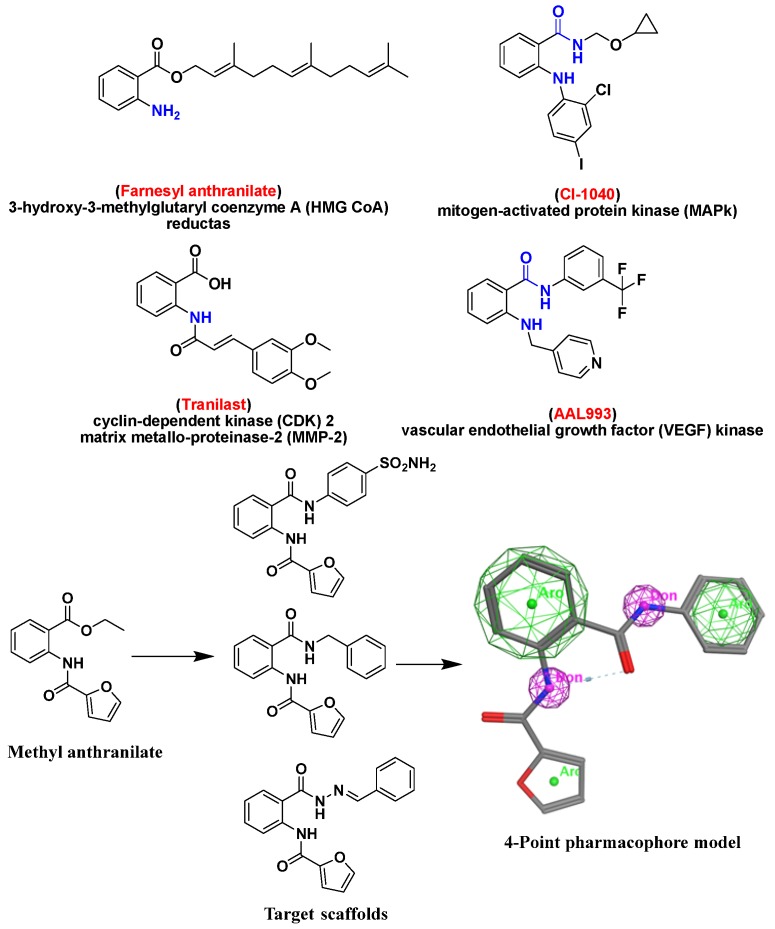
4-Point pharmacophore model of the target compounds shows the pharmacophoric group that formed from two aromatic rings and two hydrogen bond donors amino groups (2NH) surrounded by 4 electronic networks having different polarity. Green color indicates low polar area, pink color indicates high polar area and blue color indicates mild polar area.

Cancer represents a major health problem and afflicts millions of people worldwide [1,2]. Today, anticancer chemotherapy is still the main method applied in the treatment of cancer. Despite the nonstop discovery of new anticancer drugs, the treatment process is still inadequate and unsatisfactory due to several toxic effects on the normal human body cells combined with cell resistance to anticancer drugs [3,4]. Consequently, there is a vital need for discovery and development of a new class of anticancer drugs with special characteristics that are not currently used. These new classes should have less toxicity and more ability to fight the growing of drug resistance [5]. Development of drug and multidrug-resistance (MDR) represents a major problem in which cancer cells become concurrently resistant to different types of chemotherapeutic agents [6,7,8,9]. Hence, the finding and identification of new safe, effective anticancer agents are a very important goal in medicinal chemistry [10,11,12], and it was necessary to try new chemical entities as potential chemotherapeutic agents with another mechanism of action. Diamides derivatives received important attention for their antitumor activities, particularly for those having a basic diamide scaffold and pharmacophores [11]. Moreover, the anthranilic acid-based diamides derivatives have garnered significant attention for many years with a wide range of pharmacological activities [13,14] especially, anticancer activity [7,15,16,17]. Al-Obaid et al. [16] reported a molecular pharmacophore model consisting of two lipophilic aromatic points with spacer including hydrogen bond donor in rigid or flexible scaffolds that presented high activity. Taken together, our flexible design was built to comprise this molecular model and improve it with more hydrogen bond donors amino group (NH). A 4-point pharmacophore model was recognized, with two aromatic and two hydrogen bond donors (Hyd-Don-Hyd-Don) with distance ranging between each point from 3.3–7.1 Å that increase the chance for more target interactions, compound flexibility and stability. Addition of furoyl anthranilide with different amine derivatives like sulphonamides, benzylamines, and imines producing new flexible derivatives **4a**–**e**, **6a**–**c** and **9a**–**d**. Additionally, presence of heterocyclic aromatic moiety such as thiazole in compound (**6c**) will add unique spectroscopic properties to such a molecule which is known as the dual fluorescence effect. This effect makes the spectroscopic features of the compound hardly associated with excited-state intramolecular proton transfer (ESIPT) or twisted internal charge transfer (TICT) effect [18,19,20]. That may lead to improvement of their anticancer activity with good pharmacokinetic features (Figure 1). Molecular docking study is very useful to help understand binding interactions of the active analogs with their binding site through attaining ligand-receptor complex with optimized conformation and with the intention of possessing less binding free energy.

## 2. Results and Discussion

### 2.1. Chemistry

**Scheme 1:**
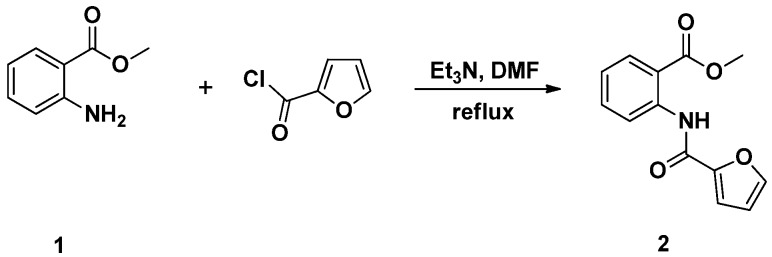
Synthesis of methyl 2-(furan-2-carboxamido) benzoate (**2**).

Acylation of methyl 2-aminobenzoate (**1**) with furoyl chloride, in the presence of triethylamine as catalyst in refluxing dimethylformamide (DMF), afforded the desired methyl 2-(furan-2-carboxamido) benzoate (**2**) in 85% yield (Scheme 1).

The success of the acylation reaction has been confirmed by IR, ^1^H neuclear magnetic resonance (NMR) and ^13^C neuclear magnetic resonance (NMR) analysis of compound (**2**). Its ^1^H neuclear magnetic resonance (NMR) spectrum showed the disappearance of the amino protons and the appearance of a downfield singlet at 11.8 parts per million (ppm) characteristic of the amidic proton NHCO. In addition, three extra aromatic protons related to the furan ring were also detected in the aromatic region at 6.7–8.6 ppm. The signal belonging to the carbonyl amide carbon resonated at 168.4 ppm in the ^13^C neuclear magnetic resonance (NMR) spectrum of compound (**2**).

**Scheme 2:**
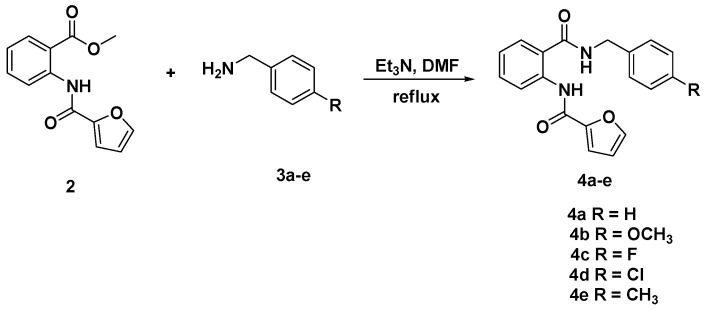
Synthesis of *N*-(2-(benzylcarbamoyl)phenyl)furan-2-carboxamide derivatives **4a**–**e**.

Nucleophilic displacement of the alkoxy group in compound (**2**) by the amino benzyl group was carried out through the reaction of compound (**2**) with different benzylamine derivatives **3a**–**e**. The reaction required thermal heating in dry dimethylformamide in the presence of catalytic amount of triethylamine and led to formation of *N*-(2-(benzylcarbamoyl)phenyl)furan-2-carboxamide **4a**–**c** in 70–75% yields (Scheme 2).

Structural elucidation of the structures of compounds **4a**–**e** has also been established on the basis of their spectral data. In their ^1^H nuclear magnetic resonance (NMR) spectra, the appearance of one distinct singlet at 3.9 ppm attributable to benzylic CH_2_ protons supported the proposed structures for compounds **4a**–**e**. The aromatic protons and carbons resonated at their appropriate aromatic region.

**Scheme 3:**
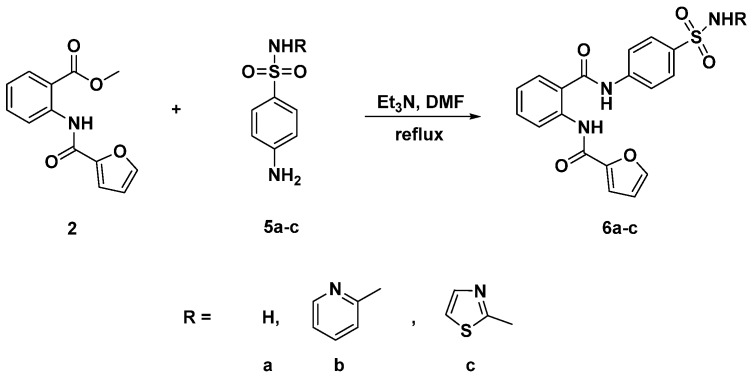
Synthesis of *N*-(2-((4-substitutedbenzenesulfamoylphenyl)carbamoyl)phenyl)furan-2-carboxamide **6a**–**c**.

Under the same optimized basic reaction conditions (triethylamine (Et_3_N), dimethylformamide (DMF)), new anthranilate diamides based-sulfonamide **6a**–**c** were designed and synthesized through the condensation of compound (**2**) with different sulfa-drugs including sulfapyridine, sulfathiazole and/or sulfonamide (Scheme 3). Elucidation of the structures of products **6a**–**c** were unambiguously confirmed by spectroscopic analysis infra-red (IR), ^1^H nuclear magnetic resonance (NMR), ^13^C nuclear magnetic resonance (NMR) and high-resolution mass (HR-MS). In their ^1^H NMR spectra, the absence of the ethoxy protons and the presence of two new exchangeable singlets at 11.5 and 10.8 attributed to the amide CONH and sulfonamide SO_2_NH protons, respectively, confirmed the incorporation of sulfa drug scaffold (moiety) in compounds **6a**–**c**. Moreover, additional aromatic protons characteristic of sulfa drugs were also observed in the aromatic region.

**Scheme 4:**
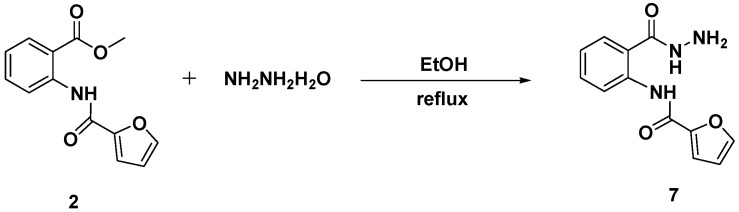
Synthesis of *N*-((2-(hydrazinecarbonyl)phenyl)furan-2-carboxamide (**7**).

Hydrazinolysis of the newly designed ester (**2**) in refluxing ethanol for 8 h afforded the targeted *N*-(2-hydrazinecarbonyl)phenyl)furan-2-carboxamide (**7**) in 95% yield (Scheme 4).

The formation of the acid hydrazide (**7**) has been clearly evidenced by ^1^H NMR and ^13^C NMR analysis through the disappearance of the ethyl ester protons and carbons. In addition, the ^1^H NMR spectrum revealed the appearance of two diagnostic singlets at 4.7 and 12.4 ppm assigned to the hydrazide NH_2_ and NH protons, respectively.

**Scheme 5:**
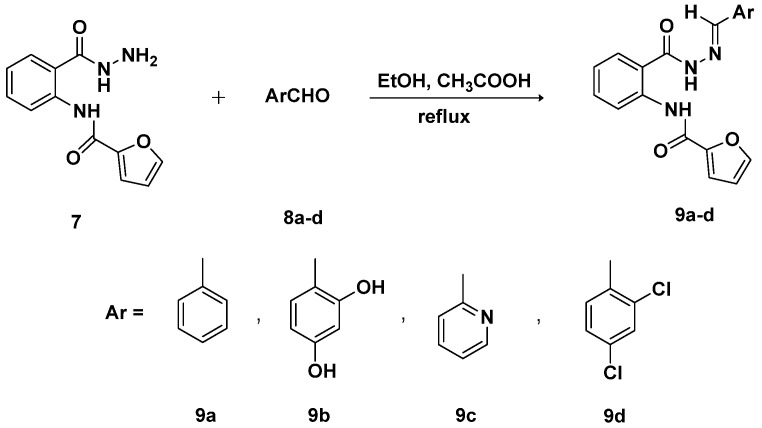
Synthesis of Schiff bases **9a**–**d**.

In the present work, new schiff bases were synthesized in good yields 95% yield by heating under reflux the acid hydrazide (**7**) with appropriate aromatic aldehydes in the presence of ethanol as solvent and acetic acid as catalyst (Scheme 5).

The structures of Schiff bases **9a**–**d** were established on the basis of their spectral data IR, ^1^H NMR, ^13^C NMR and HR-MS. The disappearance of the amino hydrazide protons and appearance of one singlet characteristic for the imine proton H–C=N at 8.5 ppm was a clear evidence for the formation of Schiff bases **9a**–**d**. In their ^13^C NMR spectra, the imine carbon H–C=N appeared in the downfield region at 146.6 ppm.

### 2.2. Cytotoxicity Screening

**Figure 2:**
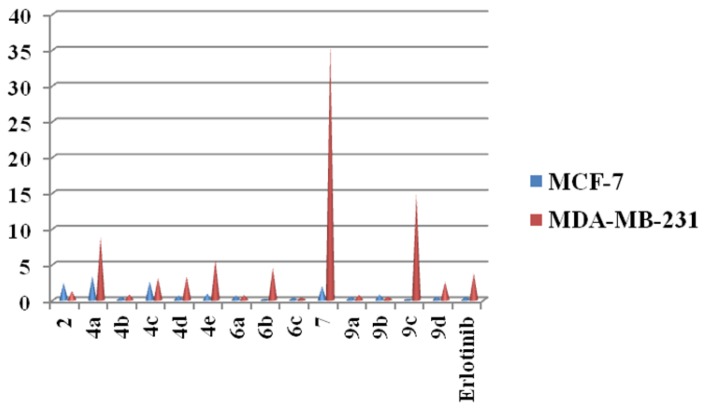
Pyramid graph for cytotoxic data of the target compounds and control drug against two cancer cell lines.

The newly synthesized compounds **4a**–**e**, **6a**–**c** and **9a**–**d** were evaluated for their in vitro anticancer activity against two breast cancer cell lines; Michigan cancer foundation-7 (MCF-7) and M.D. Anderson metastasis breast cancer-231 (MDA-MB-231) according to (3-(4,5-dimethylthiazole-2-yl)-2,5-diphenyltetrazolium bromide) tetrazolium (MTT) assay method by using erlotinib as positive tyrosine kinase (TK) drug. The half maximal inhibitory concentration (IC_50_) values are summarized in Table 1 and Figure 2. The results of in vitro anticancer activity revealed that, most of the anthranilate diamides compounds displayed excellent activity with nanomolar or subnanomolar IC_50_ values against tumor cells. The compounds bearing anthranilate diamides compounds **4a**–**e**, **6a**–**c** and **9a**–**d** showed good anticancer activity from IC_50_ 3.35 ± 0.11 to 0.065 ± 0.32 nM against the cell lines. Moreover, the compounds **9a**–**d** that bears terminal Schiff fragments exhibited the best antiproliferative effect compared to the others and control drug. In similar manner, the derivatives **6a**–**c** with sulphonamide parts showed significant cytotoxic effect against cell lines.

### 2.3. Epidermal Growth Factor Receptor (EGFR) Tyrosine Kinase (TK) Binding Inhibitory Assay

As the target compounds had potent antiproliferative activity, in vitro epidermal growth factor receptor (EGFR) inhibition capability was firstly performed for selective compounds (**4b**), (**6a**), (**6c**), (**9a**) and (**9b**) to see whether their cytotoxicity was mediated by kinase inhibition comparing to the reference staurosporine. As shown in Table 2, almost all the compounds displayed potent EGFR inhibition with IC_50_ in nanomolar range of 110 ± 0.14 to 30 ± 0.25 nM. The compounds (**4b**) and (**9b**) displayed the most potent activities IC_50_ = 31 ± 0.12 nM among all compounds (including positive control). Some compounds (**6a**) and (**9a**) exhibited similar activity as positive control drug. The compound (**4b**) bears flexible benzylamine terminal moiety, while the (**6a**) and (**6c**) derivatives bear sulphonamide moieties and **9a**–**b** with Schiff fragments. This difference in the terminal part is the key of the EGFR activity. This assumption was studied through the docking simulation. Finally, these results explained how the compounds are strong EGFR inhibitors and can probably be used as anticancer agents.

### 2.4. Effects on Tubulin Polymerization

Microtubule-targeting agents affect microtubule function and stop mitosis, resulting in necrosis and apoptosis and eventually cell death [21,22]. Initially, the same list of the EGFR tested compounds were screened for their tubulin inhibition activity IC_50_ values were reported in Table 2 and were compared with positive control colchicine. As shown in the Table 2, all examined compounds efficiently inhibited tubulin polymerization in a dose-dependent manner with IC_50_ range of 87.6 ± 0.13 to 410.6 ± 0.12 nM while compound (**6a**) turning out to be most potent nanomolar inhibitor. These results stipulate that the indicative molecular target of these analogues might be tubulin. These data explain that these compounds exhibit strong anti-tubulin polymerization activity that agrees well with their cytotoxicity.

### 2.5. Apoptosis Analysis

**Figure 3:**
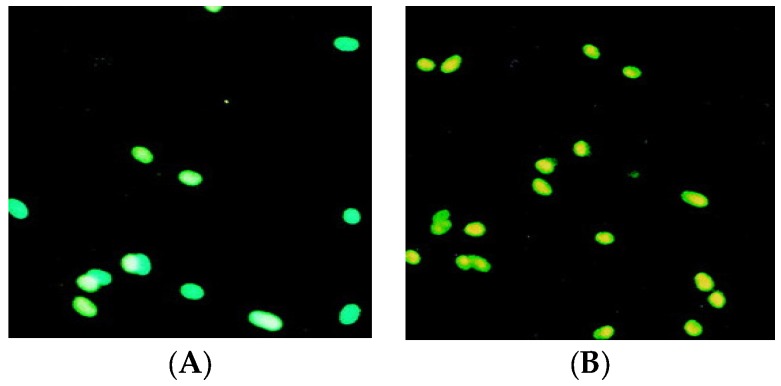
MDA-MB-231 cells with the IC_50_ concentration of compound and control drug (0.4 and 3.74 nM). After 48 h: (**A**) treated cells with erlotinib; (**B**) treated cells with (**6c**) compound displaying the apoptotic effect on the cells. Green color indicates normal cell and yellow color indicates apoptotic cell.

Apoptosis induction was examined in treated MDA-MB-231 cells by use of an Annexin V-fluorescine isothiocyanate (FITC) (Abcam, Milton, Cambridge, UK). The treatment of the cells with target compound (**6c**) and erlotinib drug with antiproliferative detected concentrations have been applied for comparison. MDA-MB-231 cells were selected as a model system because they exhibit highly metastatic and aggressive behavior in vivo compared with other human breast cancer cell lines. The apoptosis-inducing effect of target compound was investigated by flow cytometric analysis of MDA-MB-231 cells stained with Annexin V and propidium iodide. Exposure of cells to drugs for 48 h resulted in an accumulation of apoptotic cells (Figure 3). This might be extra behavior interpreting the potent cytotoxic effect of such compounds.

### 2.6. Molecular Docking Studies and Structure Activity Relationship (SAR) Analyses

**Figure 4:**
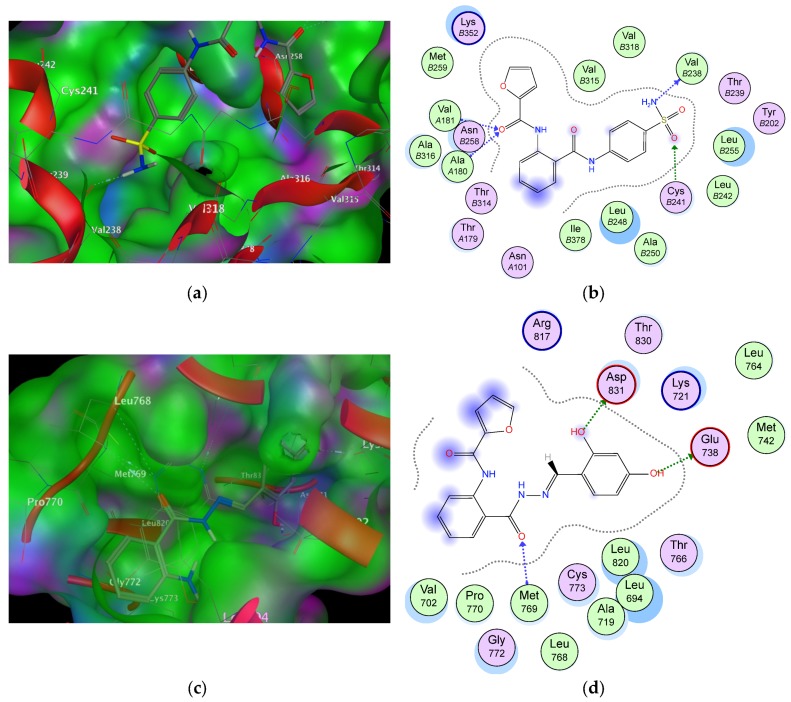
The top images (**a**,**b**) show 3D and 2D graphs of compound (**9b**) docked into colchicine binding site of protein (1SA0). The bottom images (**c**,**d**) show 3D and 2D graphs of compound (**6a**) docked into ATP binding site of protein (1M17). Green color indicates hydrophobic area, pink color indicates high polar area, blue color indicates mild polar area and dotted lines and arrows represent hydrogen bonds.

To rationalize the experimental results obtained, molecular docking studies were accomplished on representative compounds (**6a**) and (**9b**) against two targets, EGFR and tubulin in colchicine binding site structures. It is well known that the colchicine binding site is generally present at the interface of α, and β-tubulin heterodimers. It was clear that the all compound fragments share in the interaction with the binding pockets. This means the activity of such series are related to both scaffold and terminal fragments. From the data reported in Table 3 and regarding to tubulin target, the compound (**6a**) showed critical interactions between Val238 and Cys241 and sulphonamide and anthranilate fragments. The key hydrogen bonds formed due to the SO_2_NH_2_ of the compound and two corresponding residues of the colchicine binding pocket (Figure 4a,b). Moreover, the anthranilate C=O scaffold formed a stable hydrogen bond with the two hydrophobic amino acids, Val181 and Ala180. The structure activity relationship (SAR) analysis revealed the importance of the terminal sulphonamide moiety. The behavior of compound (**9b**) as potent EGFR inhibitor in the binding pocket showed consistently stable two hydrogen bonds with the terminal hydroxyl groups. One of the most interactions of the compound is the extra hydrogen bond between the C=O of the linker and the conserved amino acid Met769 (Figure 4c,d). All the modeled drugs displayed hydrophobic interactions (hydrocarbons-hydrocarbons) between the hydrophobic groups in the amino acids residues and the hydrophobic parts in the ligands. That stabilized the compounds in the binding pockets. At the end, the scaffold, linker, terminal fragments are essential for activity and it is very important to take care during any optimization processes.

## 3. Materials and Methods

### 3.1. Chemistry

Melting points were carried out using a Mel-Temp melting point apparatus and all melting points are uncorrected. Infrared spectra were recorded in pottasium bromide (KBr) disc on a Schimadzu 8201, spectrophotometer (Schimadzu, Chiyoda-ku, Tokyo, Japan) (ν_max_ in cm^−1^). ^1^H NMR and ^13^C NMR spectra were recorded on a Bruker AC 400 MHz NMR spectrometer (Bruker, Madison, WI, USA) at 400 for ^1^H and 100 MHz for ^13^C respectively. All ^1^H and ^13^C NMR spectral data are recorded as chemical shifts (d). Chemical shifts were measured in DMSO-*d_6_* and were relative to the solvent peak of 2.5 ppm for ^1^H spectra and 39.5 ppm for ^13^C spectra. Complete scan precise mass spectra (mass range from 200 to 1500 Da) were gained at high resolution (100,000, full-width half-maximum (FWHM) at 400 *m*/*z* on a LTQ-FT Orbitrap Velos mass spectrometer (Thermo Electron GmbH, Freiburg, Germany). The hybrid mass spectrometer contains heated electrospray ionization (ESI) probe unit. The electrospray source conditions were: capillary voltage 3.75 kV, heated at 275 °C, source temperature 450 °C and S-Lens rear focusing (RF) level 60%. The flows of nitrogen gas were set at 20–40. Mass spectra were determined on a Shimadzu GC/MS-QP5050A spectrometer (Schimadzu, Chiyoda-ku, Tokyo, Japan). Elemental analyses were carried out at the Regional Centre for Mycology & Biotechnology (RCMP), Al-Azhar University, Cairo, Egypt and the results were within ±0.25%. Reaction courses and product mixtures were routinely monitored by thin layer chromatography (TLC) on silica gel pre coated F_254_ Merck plates.

Study of the pharmacophoric model of the previously reported anthranilate diamide anticancer derivatives helped us to design new derivatives of these compounds through addition of furoyl anthranilide with different amine derivatives like sulphonamides, benzylamines and imines. These compounds were selected to produce new derivatives **4a**–**e**, **6a**–**c** and **9a**–**d** maintaining the required pharmacophoric features (two aromatic rings and two NH groups) and having a different electronic environment with a more flexible pattern and characterized spectroscopic features which may lead to improvement of their anticancer activity profile.

### 3.2. Synthesis and Characterization of Methyl 2-(Furan-2-carboxamido)benzoate *(**2**)*

To a stirring solution of methyl-2-aminobenzoate (**1**) (1 mmol) and triethylamine (5 mmol) in dry DMF (10 mL), 2-furoyl chloride (1.2 mmol) was then added dropwise. The reaction mixture was heated under reflux for 4 h. After cooling, the reaction mixture was poured into ice water and the formed solid was collected by filtration and recrystallized from ethanol to give (**2**) (85% yield), melting point (mp) 190–192 °C. ν_max_ (KBr)/cm^−1^ 3368, 2965 (N–H), 1720 (C=O), 1693 (C=O); ^1^H NMR (DMSO-*d*_6_) δ_H_ 11.8 broad-singlet (bs, 1H, CONH), 6.7–8.6 multiplet (m, 7H, Ph-H and furoyl-H), 3.9 singlet (s, 3H, OCH_3_); ^13^C NMR (DMSO-*d*_6_) δ_C_ 168.4, 156.2, 147.7, 146.7, 140.6, 135.0, 131.3, 123.7, 120.6, 116.3, 116.2, 113.3 (Ar-H, C=O), 53.20 (CH_3_); HR-MS (ESI) *m*/*z*: calculated for C_13_H_11_NO_4_ (M + H)^+^: 245.0688, found: 245.0679.

### 3.3. General Procedure for the Synthesis of Diamides ***4a**–**e*** and ***6a**–**c***

A mixture of the appropriate benzylamine and/or sulphonamide derivatives (1.5 mmol), triethylamine (6.5 mmol) in DMF (10 mL) was stirred under reflux for 2 h, a solution of methyl 2-(furan-2-carboxamido)benzoate (**2**) (1 mmol) in DMF (10 mL) was added portion-wise. The reaction mixture was refluxed for overnight until the reaction was judged complete by TLC and then poured into ice water. The resulting solid thus formed was filtered and recrystallized from the appropriate solvent.

#### 3.3.1. *N*-[(2-(Benzylcarbamoyl)phenyl]furan-2-carboxamide (**4a**)

This compound was obtained in 75% yield (from ethyl acetate), mp 230–232 °C. ν_max_ (KBr)/cm^−1^ 3358, 2955 (N–H), 1710 (C=O), 1698 (C=O); ^1^H NMR (DMSO-*d*_6_) δ_H_ 10.2 (bs, 1H, NHCO), 7.1–8.2 (m, 12H, Ph-H, furoyl-H and benzyl-H), 7.3 (bs, 1H, NHCH_2_), 3.9 (s, 2H, CH_2_); ^13^C NMR (DMSO-*d*_6_) δ_C_ 167.8, 162.2, 148.7, 143.2, 138.6, 133.7, 131.6, 125.4, 126.9, 126.6, 125.0, 122.6, 115.3, 114.2, 111.7 (Ar-C, C=O), 44.1 (CH_2_), HR-MS (ESI) *m*/*z*: calculated for C_19_H_16_N_2_O_3_ (M + H)^+^: 320.1161, found: 320.1158.

#### 3.3.2. *N*-{2-[(4-Methoxybenzyl)carbamoyl]phenyl}furan-2-carboxamide (**4b**)

This compound was obtained in 73% yield (from ethanol), mp 207–209 °C. ν_max_ (KBr)/cm^−1^ 3368, 2965 (N–H), 1715 (C=O), 1695 (C=O); ^1^H NMR (DMSO-*d*_6_) δ_H_ 10.6 (bs, 1H, NHCO), 6.8–8.3 (m, 11H, Ph-H, furoyl-H and benzyl-H), 7.2 (bs, 1H, NHCH_2_), 3.9 (s, 2H, CH_2_), 3.7 (s, 3H, O-CH_3_); ^13^C NMR (DMSO-*d*_6_) δ_C_ 167.3, 161.6, 158.6, 148.2, 143.8, 137.6, 132.7, 131.8, 130.4, 125.0, 124.6, 119.5, 115.8, 114.5, 112.3 (Ar-C, C=O), 55.6 (OCH_3_), 44.1 (CH_2_), HR-MS (ESI) *m*/*z*: calculated for C_20_H_18_N_2_O_4_ (M + H)^+^: 350.1267, found: 350.1261.

#### 3.3.3. *N*-{2-[(4-Fluorobenzyl)carbamoyl]phenyl}furan-2-carboxamide (**4c**)

This compound was obtained in 75% yield (from ethanol), mp 218–220 °C. ν_max_ (KBr)/cm^−1^ 3358, 2955 (N–H), 1725 (C=O), 1695 (C=O); ^1^H NMR (DMSO-*d*_6_) δ_H_ 10.5 (bs, 1H, NHCO), 7.1–8.2 (m, 11H, Ph-H, furoyl-H and benzyl-H), 7.3 (bs, 1H, NHCH_2_), 3.9 (s, 2H, CH_2_); ^13^C NMR (DMSO-*d*_6_) δ_C_ 167.8, 162.7, 160.6, 148.2, 143.5, 137.9, 133.2, 132.3, 131.6, 128.4, 125.0, 124.6, 118.5, 115.4, 111.7 (Ar-C, C=O), 44.1 (CH_2_), HR-MS (ESI) *m*/*z*: calculated for C_19_H_15_FN_2_O_3_ (M + H)^+^: 338.1067, found: 338.1062.

#### 3.3.4. *N*-{2-[(4-Chlorobenzyl)carbamoyl]phenyl}furan-2-carboxamide (**4d**)

This compound was obtained in 72% yield (from ethanol), mp 213–215 °C. ν_max_ (KBr)/cm^−1^ 3358, 2955 (N–H), 1710 (C=O), 1698 (C=O); ^1^H NMR (DMSO-*d*_6_) δ_H_ 10.6 (bs, 1H, NHCO), 7.1–8.2 (m, 11H, Ph-H, furoyl-H and benzyl-H), 7.3 (bs, 1H, NHCH_2_), 3.9 (s, 2H, CH_2_); ^13^C NMR (DMSO-*d*_6_) δ_C_ 168.2, 161.9, 160.2, 148.2, 143.7, 138.2, 133.8, 131.7, 131.6, 128.4, 125.6, 123.3, 117.5, 115.8, 111.2 (Ar-C, C=O), 44.1 (CH_2_), HR-MS (ESI) *m*/*z*: calculated for C_19_H_15_ClN_2_O_3_ (M + H)^+^: 354.0771, found: 354.0778.

#### 3.3.5. *N*-{2-[(4-Methylbenzyl)carbamoyl]phenyl}furan-2-carboxamide (**4e**)

This compound was obtained in 70% yield (from ethanol), mp 175–178 °C. ν_max_ (KBr)/cm^−1^ 3358, 2955 (N–H), 1713 (C=O), 1688 (C=O); ^1^H NMR (DMSO-*d*_6_) δ_H_ 10.5 (bs, 1H, NHCO), 6.9–8.4 (m, 11H, Ph-H, furoyl-H and benzyl-H), 7.2 (bs, 1H, NHCH_2_), 4.0 (s, 2H, CH_2_), 2.3 (s, 3H, CH_3_); ^13^C NMR (DMSO-*d*_6_) δ_C_ 167.8, 162.7, 160.6, 149.2, 142.5, 138.1, 133.2, 132.3, 131.6, 129.4, 125.5, 124.9, 118.9, 114.8, 112.1 (Ar-C, C=O), 44.5 (CH_2_), 21.5 (CH_3_), HR-MS (ESI) *m*/*z*: calculated for C_20_H_18_N_2_O_3_ (M + H)^+^: 334.1317, found: 334.1311.

#### 3.3.6. *N*-{2-[(4-Sulfamoylphenyl)carbamoyl]phenyl}furan-2-carboxamide (**6a**)

This compound was obtained in 75% yield (from 1,4-dioxan), mp 188–189 °C. ν_max_ (KBr)/cm^−1^ 3357, 3203 (N–H), 1678 (C=O), 1632 (C=O), 1341, 1152 (S=O); ^1^H NMR (DMSO-*d*_6_) δ_H_ 11.5 (bs, 1H, NHCO), 10.2 (bs, 1H, NHCO), 8.6–6.7 (m, 11H, Ph-H, furoyl-H and aminosulphonyl ph-H), 3.4 (bs, 2H, NH_2_); ^13^C NMR (DMSO-*d*_6_) δ_C_ 168.2, 162.9, 148.2, 143.5, 141.3, 137.2, 136.3, 132.6, 129.4, 127.7, 124.6, 123.4, 120.2, 118.5, 115.9, 112.1 (Ar-C, C=O), HR-MS (ESI) *m*/*z*: calculated for C_18_H_15_N_3_O_5_S (M + H)^+^: 385.0732, found: 385.0741.

#### 3.3.7. *N*-{2-[(4-(*N*-(Pyridin-2-Yl)sulfamoyl]phenyl)carbamoyl)phenyl}furan-2-carboxamide (**6b**)

This compound was obtained in 64% yield (from acetonitraile), mp 209–211 °C. ν_max_ (KBr)/cm^−1^ 3357, 3203 (N–H), 1675 (C=O), 1640 (C=O), 1341, 1152 (S=O); ^1^H NMR (DMSO-*d*_6_) δ_H_ 11.3 (bs, 1H, NHCO), 10.8 (bs, 1H, SO_2_NH), 10.5 (bs, 1H, NHCO), 6.7–8.6 (m, 15H, Ph-H, furoyl-H and aminosulphonyl ph-H, pyridinyl-H); ^13^C NMR (DMSO-*d*_6_) δ_C_ 167.1, 162.4, 148.1, 143.4, 141.2, 137.5, 135.3, 132.6, 129.7, 127.9, 124.1, 123.2, 119.5, 118.2, 115.6, 111.3, 109.5 (Ar-C, C=O), HR-MS (ESI) *m*/*z*: calculated for C_23_H_18_N_4_O_5_S (M + H)^+^: 462.0998, found: 462.0992.

#### 3.3.8. *N*-{2-[(4-(*N*-(Thiazol-2-Yl)sulfamoyl)phenyl)carbamoyl]phenyl}furan-2-carboxamide (**6c**)

This compound was obtained in 62% yield (from ethanol), mp 217–219 °C. ν_max_ (KBr)/cm^−1^ 3348, 3205 (N–H), 1681 (C=O), 1642 (C=O), 1341, 1152 (S=O); ^1^H NMR (DMSO-*d*_6_) δ_H_ 11.2 (bs, 1H, NHCO), 10.8 (bs, 1H, SO_2_NH), 10.3 (bs, 1H, NHCO), 6.3–8.6 (m, 13H, Ph-H, furoyl-H and aminosulphonyl ph-H, thiazole-H); ^13^C NMR (DMSO-*d*_6_) δ_C_ 170.4, 167.3, 161.7, 147.9, 143.8, 140.7, 137.9, 136.8, 134.6, 133.1, 129.4, 128.1, 123.8, 122.7, 119.2, 118.0, 116.1, 113.1, 111.7 (Ar-C, C=O), HR-MS (ESI) *m*/*z*: calculated for C_23_H_18_N_4_O_5_S (M + H)^+^: 462.0998, found: 462.0991.

### 3.4. Synthesis and Characterization of N-[2-(Hydrazinecarbonyl)phenyl]furan-2-carboxamide *(**7**)*

A mixture of methyl-2-(furan-2-carboxamido)benzoate (**2**) (1 mmol) and hydrazine hydrate (5 mmol) in ethanol (20 mL) was refluxed for 8 h. After cooling, the mixture was poured into crushed ice and the formed solid was filtered off. The crude product was recrystallized from hot water to give compound (**7**) in 95% yield, mp 210–212 °C. ν_max_ (KBr)/cm^−1^ 3204–3210 (NH_2_, NH), 1689 (C=O), 1657 (C=O); ^1^H NMR (DMSO-*d*_6_) δ_H_ 12.4 (bs, 1H, NHNH_2_), 10.2 (bs, 1H, NHCO), 6.7–8.6 (m, 7H, Ph-H and furoyl-H), 4.7 (s, 2H, NH_2_); ^13^C NMR (DMSO-*d*_6_) δ_C_ 168.0, 156.1, 148.0, 146.5, 139.1, 132.5, 128.3, 123.7, 120.6, 119.5, 115.9, 113.0 (Ar-C, C=O); HR-MS (ESI) *m*/*z*: calculated for C_12_H_11_N_3_O_3_ (M + H)^+^: 245.0800, found: 245.0807.

### 3.5. General Procedure for the Synthesis of Schiff Bases-Type Hydrazones ***9a**–**d***

A mixture of *N*-[2-(hydrazinecarbonyl)phenyl]furan-2-carboxamide (**7**) (1 mmol) and the appropriate aromatic aldehyde **8a**–**d** (1 mmol) in 20 mL of ethanol and few drops of glacial acetic acid was refluxed for 3 h. After cooling, the reaction mixture was poured into ice water, and the formed solid product was filtered. The crude product was recrystallized from the appropriate solvent.

#### 3.5.1. (*E*)-*N*-{2-[(2-Benzylidenehydrazine)-1-carbonyl]phenyl}furan-2-carboxamide (**9a**)

This compound was obtained in 60% yield (from ethanol), mp 205–207 °C. ν_max_ (KBr)/cm^−1^ 3248, 2895 (N–H), 1697 (C=O), 1676 (C=O), 1621 (N=N); ^1^H NMR (DMSO-*d*_6_) δ_H_ 11.7 (bs, 1H, CONH), 11.3 (bs, 1H, CONH), 6.7–8.5 (m, 13H, Ph-H, furoyl-H, phenyl-H and H–C=N); ^13^C NMR (DMSO-*d*_6_) δ_C_ 166.2, 154.7, 152.0, 150.8, 148.5, 146.6, 143.7, 138.7, 137.9, 134.4, 130.3, 129.7, 128.4, 125.7, 122.5, 121.7, 120.8, 116.5, 112.6 (Ar-C, C=O, C=N); HR-MS (ESI) *m*/*z*: calculated for C_19_H_15_N_3_O_3_ (M + H)^+^: 333.1113, found: 333.1108.

#### 3.5.2. (*E*)-*N*-{2-[2-(2,4-Dihydroxybenzylidene)hydrazine-1-carbonyl]phenyl}furan-2-carboxamide (**9b**)

This compound was obtained in 60% yield (from ethanol), mp 215–217 °C. ν_max_ (KBr)/cm^−1^ 3380, 3252 (O–H), 3248, 2895 (N–H), 1697 (C=O), 1676 (C=O), 1597 (N=N); ^1^H NMR (DMSO-*d*_6_) δ_H_ 11.8 (bs, 1H, CONH), 11.3 (bs, 1H, OH), 10.5 (bs, 1H, CONH), 9.3 (bs, 1H, OH), 6.4–8.6 (m, 13H, Ph-H, furoyl-H, phenyl-H, and H–C=N); ^13^C NMR (DMSO-*d*_6_) δ_C_ 164.8, 162.7, 162.3, 161.7, 148.2, 146.5, 143.8, 138.0, 133.5, 132.7, 127.7, 124.5, 123.7, 119.4, 115.3, 112.6, 111.5, 108.3, 103.4 (Ar-C, C=O, C=N); HR-MS (ESI) *m*/*z*: calculated for C_19_H_15_N_3_O_5_ (M + H)^+^: 365.1012, found: 365.1019.

#### 3.5.3. (*E*)-*N*-{2-[2-(Pyridin-2-ylmethylene)hydrazine-1-carbonyl]phenyl}furan-2-carboxamide (**9c**)

This compound was obtained in 65% yield (from ethyl acetate), mp 225–227 °C. ν_max_ (KBr)/cm^−1^ 3238, 2905 (N–H), 1705 (C=O), 1667 (C=O), 1620 (N=N); ^1^H NMR (DMSO-*d*_6_) δ_H_ 12.3 (bs, 1H, CONH), 11.7 (bs, 1H, CONH), 6.7–8.6 (m, 12H, Ph-H, furoyl-H, pyridyl-H and H–C=N); ^13^C NMR (DMSO-*d*_6_) δ_C_ 165.4, 156.2, 153.3, 150.1, 149.7, 147.7, 146.6, 139.0, 137.3, 133.2, 129.1, 125.0, 123.7, 121.2, 120.7, 120.6, 116.0, 113.2 (Ar-C, C=O, C=N); HR-MS (ESI) *m*/*z*: calculated for C_23_H_18_N_4_O_5_S (M + H)^+^: 462.0998, found: 462.0989.

#### 3.5.4. (*E*)-*N*-{2-[2-(2,4-Dichlorobenzylidene)hydrazine-1-carbonyl]phenyl}furan-2-carboxamide (**9d**)

This compound was obtained in 60% yield (from toluene), mp 212–214 °C. ν_max_ (KBr)/cm^−1^ 3248, 2895 (N–H), 1697 (C=O), 1676 (C=O), 1597 (N=N); ^1^H NMR (DMSO-*d*_6_) δ_H_ 11.8 (bs, 1H, CONH), 11.3 (bs, 1H, CONH), 6.4–8.6 (m, 11H, Ph-H, furoyl-H, phenyl-H and H–C=N); ^13^C NMR (DMSO-*d*_6_) δ_C_ 165.4, 156.1, 148.2, 143.8, 138.0, 137.3, 133.7, 132.8, 131.5, 129.7, 129.5, 128.3, 127.7, 127.0, 124.4, 123.2, 119.4, 115.5, 111.7 (Ar-C, C=O, C=N); HR-MS (ESI) *m*/*z*: calculated for C_19_H_13_Cl_2_N_3_O_5_ (M + H)^+^: 433.0232, found: 433.0226.

### 3.6. Cancer Cell Antiproliferative Assay

The in vitro anticancer activities of the target compounds against two breast cancer cell lines; luminal human breast adenocarcinoma (estrogen-receptor (ER) and progesterone-receptor (PR)) positive MCF-7 and negative human breast cancer (MDA-MB-231) cell lines were evaluated as described with some modifications [23]. Cell line cells were obtained from American Type Culture Collection, cells was cultured using DMEM (Invitrogen Life Technologies, Waltham, MA, USA) supplemented with 10% FBS (Hyclone, Logan, UT, USA), 10 µg/mL of insulin (Sigma, St. Louis, MO, USA), and 1% penicillin-streptomycin. All of the other chemical sand reagents were from (Sigma, St. Louis, MO, USA). The cells (Hs 578T) were sub-cultured into a 96-well plate with 1104 cells per well in medium, at 37 °C, 5% CO_2_ and 95% air atmosphere, before being treated with or without various concentrations of test compounds, each in triplicate for 24 h. At the end of the incubation, the cells were harvested and washed with PBS. 20 µL of MTT was added to each well and incubated for 2 h before 200 µL DMSO was added. The absorbance was measured on an ELISA reader (Multiskan EX, Lab systems, Waltham, MA, USA) at a test wavelength of 570 nm [24].

### 3.7. EGFR Inhibition Assay

EGFR kinase activity was measured by HT Scan EGFR kinase assay kits (Cell Signaling Technology, Danvers, MA, USA). The experiments were done following the manufacturer’s directions. In conclusion, Synthetic peptide substrate, 10 μg/mL inhibitors and the glutathione s-transferase (GST)-EGFR protein were incubated in the presence of 400 μM ATP. Strapavidin-coated 96-well plates was used to take phosphorylated substrate. Anti-phosphotyrosine and europium-labeled secondary antibodies (DELFIA, Perkin-Elmer, Akron, OH, USA) was used to monitor the level of phosphorylation. At the end of the assay, the enrichment solution was added and enzyme activity was assessed in the Wallac Victor II 1420 micro-plate reader (Abcam, Milton, Cambridge, UK) and 1% penicillin-streptomycin. All of the other chemical sand reagents were from (Abcam, Milton, Cambridge, UK) at 615 nM.

### 3.8. Enzyme-Linked Immunosorbent Assay for Tubulin Beta (Tubb)

This assay was done according to reported procedures [21,22]

### 3.9. MDA-MB-231 Cell Line Annexin V-FITC Apoptosis Analysis

This analysis was performed following the reported procedures [25,26].

### 3.10. Molecular Docking

Molecular docking study was performed for the selected active compounds into the three-dimensional complex of two biological targets comprising the crystal structures of EGFR (PDB code: 1M17) at 2.6 Å resolution and tubulin in complex with colchicine and the stathmin-like domain (SLD) at 3.5 Å resolution (PDB: 1SA0) [27] was done using the auto dock software package (version 4.0 (Molecular graphics laboratory, La Jolla, CA, USA) as implemented through the graphical user interface auto dock tools (ADT) [25,26,27,28,29,30,31].

## 4. Conclusions

In conclusion, we have designed some new derivatives of *n*-furoyl anthranilate and evaluated their cytotoxicities in vitro against a panel of two different cancer cell lines. Most of the diamide compounds exhibited potent cytotoxic activities against tumor cells. The results of this study also revealed that diamides bearing different amines generally displayed good levels of cytotoxicity. The position and the type of the substituents had a significant impact on the cytotoxic activity. The most potent compounds were (**6a**) and (**9b**). The mechanism of action of such newly synthesized derivatives was investigated through EGFR TK binding inhibitory assay which proved strong EGFR inhibitory activity. Tubulin polymerization assay and apoptosis analysis proved the potent cytotoxic activities of these compounds. The molecular modeling study explained the potent interaction of target compounds with their binding sites. They bind in the same manner as reference drugs. Based on that, the most active compounds could be used as a pattern for future design, optimization and investigation to produce more effective analogs as potent anticancer agents.

## Figures and Tables

**Table 1:** In vitro cell proliferation of compounds anthranilate diamides compounds **4a**–**e**, **6a**–**c** and **9a**–**d** and erlotinib against MCF-7 and MDA-MB-231 breast cancer cell lines.

Compounds	IC_50_ (nM)	
	MCF-7	MDA-MB-231
**2**	2.43 ± 0.20	1.21 ± 0.25
**4a**	3.35 ± 0.11	8.82 ± 0.14
**4b**	0.45 ± 0.12	0.76 ± 0.11
**4c**	2.59 ± 0.32	3.11 ± 0.50
**4d**	0.64 ± 0.25	3.29 ± 0.14
**4e**	0.88 ± 0.25	5.59 ± 0.20
**6a**	0.62 ± 0.12	0.66 ± 0.11
**6b**	0.065 ± 0.32	4.45 ± 0.30
**6c**	0.34 ± 0.20	0.32 ± 0.21
**7**	1.91 ± 0.11	35.85 ± 0.12
**9a**	0.51 ± 0.16	0.68 ± 0.02
**9b**	0.78 ± 0.11	0.48 ± 0.01
**9c**	0.15 ± 0.15	14.8 ± 0.20
**9d**	0.48 ± 0.14	2.53 ± 0.12
Erlotinib	0.45 ± 0.08	3.74 ± 0.01

IC_50_, half maximal inhibitory concentration.

**Table 2:** Inhibitory data of the selected compounds to both epidermal growth factor receptor (EGFR) tyrosine kinase (TK) and tubulin polymerization.

Compounds	IC_50_ (nM)	
	EGFR Inhibition	Tubulin Assay
**4b**	30.5 ± 0.25	140.8 ± 0.15
**6a**	85.4 ± 0.32	87.6 ± 0.13
**6c**	110 ± 0.14	155 ± 0.25
**9a**	59.3 ± 0.11	410.6 ± 0.12
**9b**	31.2 ± 0.12	108.4 ± 0.14
Staurosporine	59.08 ± 0.2	-
Colchicine	-	112 ± 0.08

**Table 3:** Description of the docking data of selected target compounds.

Cop.	Fragment	Target Residues (Distance, Å)	Interaction	Binding Energy (dG)
**6a** (1SA0)	–NH_2_	Val238 (2.5)	H-bonding	−16.3
	S=O	Cys241 (2.00)	H-bonding	
	C=O–NH	Val181 (1.85), Ala180 (1.91)	H-bonding	
	Phenyl (sulphonamide)	Val315, Leu248, Val318	Hydrophobic	
	Furoyl	Met259	Hydrophobic	
	Phenyl (anthranilate)	Thr514, Ile378	Aromatic stacking	
**9b** (1M17)	–OH	Asp831 (2.1)	H-bonding	−15.4
	–OH	Glu738 (1.84)	H-bonding	
	C=O (anthranilate)	Met769 (2.1)	Hydrophobic	
	Phenyl (sulphonamide)	Leu820	Hydrophobic	
	Furoyl	Arg817	Hydrophobic	
	Phenyl (anthranilate)	Val702, Pro770	Hydrophobic	

The data reported in the table are extracted from molecular operating environment (MOE) program showing fragments of the two ligands, amino acids residues in the binding pocket, types of interaction and the values of their binding energy with the two targets 1SA0 and 1M17.
